# Action of Halowax 1051 on Enzymes of Phase I (CYP1A1) and Phase II (SULT1A and COMT) Metabolism in the Pig Ovary

**DOI:** 10.1155/2013/590261

**Published:** 2013-04-04

**Authors:** Justyna Barć, Anna Karpeta, Ewa Łucja Gregoraszczuk

**Affiliations:** Department of Physiology and Toxicology of Reproduction, Institute of Zoology, Jagiellonian University, 31-387 Krakow, Poland

## Abstract

Polychlorinated naphthalenes (PCNs) are a group of organochlorinated compounds exhibiting dioxin-like properties. Previously published data showed the direct action of PCN-rich Halowax 1051 on ovarian follicular steroidogenesis. Taking into consideration that the observed biological effects of PCNs may be frequently side effects of metabolites generated by their detoxification, the aim of this study was to determine the activity and expression of enzymes involved in phase I (cytochrome P450, family 1 (CYP1A1)) and phase II (sulfotransferase (SULT1A) and catechol-O-methyltransferase (COMT)) detoxification metabolism. Cocultures of granulosa and theca interna cells collected from sexually mature pigs were exposed to 1 pg/mL to 10 ng/mL of Halowax 1051 for 1 to 48 hours, after which levels and activities of CYP1A1, SULT1A, and COMT were measured. Dose-dependent increases of CYP1A1 activity and expression were observed. High doses of Halowax 1051 were inhibitory to COMT and SULT1A activity and reduced their protein levels. In conclusion, fast activation of phase I enzymes with simultaneous inhibition of phase II enzymes indicates that the previously observed effect of Halowax 1051 on follicular steroidogenesis may partially result from metabolite action occurring locally in ovarian follicles.

## 1. Introduction

Polychlorinated naphthalenes (PCNs) are chlorinated organic compounds that have been used in various industries as capacitor impregnates, electrical insulating compounds, flame-resistant seals for condensers, gauge fluids, and lubricants because of their beneficial properties [1]. Until the 1980s, PCNs as mixtures of congeners were synthesized in North America (Halowax) and Europe (e.g., Nibren Wax, Basileum, and Seekay Wax) [2]. Currently, there is no commercial use for PCNs [1]. Production of PCNs has ceased due to its substitution by less toxic chemicals [3]. Even though the production of PCNs has ended, humans are still exposed to PCNs via food consumption [4–7].

As with other polychlorinated diaromatic hydrocarbons, PCNs are lipophilic compounds that persist in the environment and bioaccumulate in biological tissues [8]. There are reports indicating the presence of PCNs in adipose tissue and body fluids of people exposed to these agents [6, 9, 10].

In spite of significant research into the presence of PCNs in various samples and their dioxin-like properties, data concerning their action as endocrine disruptors are scarce. Akerblom et al. [11] showed that differences in oocyte maturation exist between control and PCNs-exposed ovaries. Further, published data from our laboratory showed the direct action of Halowax 1051 on ovarian follicular steroidogenesis [12]. Together, these reports indicate that PCNs can disrupt the endocrine system, thereby leading to reproduction defects. 

It should be taken into consideration that the effects of exposure to PCNs may be due to the side effects of PCN metabolites. Environmental chemicals such as PCNs may be metabolized to more polar compounds in living organisms. For instance, PCNs can be transformed into hydroxylated metabolites [3]. Cytochrome P450 proteins (CYPs) play key roles in the metabolism and elimination of exogenous substances. Enzymes belonging to the CYP family are called phase I enzymes; they monooxygenate, reduce, and hydrolyze various substances such as lipids, steroidal hormones, and xenobiotics [13]. Enzymes of phase I are expressed mainly in the liver, but they are also present in other tissues such as uterus, adrenal glands, placenta, kidney, brain, and testis [14]. In previously published papers we showed that three cytochrome P450 (CYP) isoforms, CYP1A1, CYP1A2 and CYP2B, are present in porcine prepubertal ovary cells [15–17].

After phase I reactions, xenobiotics may be further metabolized by conjugation with charged species such as glutathione, sulfate, glycine, or glucuronic acid to form more polar products, which are more efficiently eliminated from the organism. Rabbits exposed to monochloro- or dichloronaphthalenes excreted 70 to 90% of these compounds in four days, mainly as conjugates of glucuronic acid (54–69%) and mercapturic acid (13–18%). Minor amounts of naphthalene sulfates and phenolic conjugates were also excreted [3]. Phase II enzymes are expressed mainly in the liver, but they are also found in the ovary. Catechol-O-methyltransferase (COMT) is expressed in porcine and human granulosa cells [17, 18] and sulfotransferase (SULT1A) is expressed in porcine ovaries [17, 19]. When determining the metabolism of 2,2′,4,4′-tetrabromodiphenyl ether (BDE-47) in the porcine ovary, Karpeta et al. [17] showed fast activation of CYP2B1/2, late activation of COMT, and lack of activation of SULT1A. This confirmed the action of phase II enzymes in the ovary and suggested the possible action of locally produced hydroxylated metabolites prior to their detoxification.

In this study, we ought to determine whether PCNs are metabolized in the ovary by examining the effects of the Halowax 1051 on phase I (CYP1A1) and phase II (SULT1A and COMT) enzyme activities and expression in cultured granulosa and theca interna cells. 

## 2. Materials and Methods

### 2.1. Reagents

Halowax 1051 was obtained from Koppers Co., USA. A stock solution of this compound was dissolved in DMSO. The final concentration of DMSO in the medium was 0.1%. Parker's medium (M199) lacking phenol red, trypan blue, Laemmli lysis-buffer, dimethyl sulfoxide (DMSO), Tris, sodium dodecyl sulfate (SDS), Tween 20, 4-nitrocatechol, SAM, 3,5-diniotrocatechol, PAPS, p-nitrophenyl sulfate, 2-naphthol, and 2,6-dichloro-4-niotrophenol were obtained from Sigma Chemical Co. (Saint Louis, MO, USA). Fetal bovine serum (FBS, heat inactivated), phosphate-buffered saline (PBS), and Trypsin-EDTA and antibiotic, antimycotic solution (penicillin 100 U/mL, streptomycin 100 *μ*g/mL, amphotericin B 0.25 *μ*g/mL) were obtained from PAA Laboratories GmbH (Colbe, Germany).

### 2.2. Tissue Culture

Porcine prepubertal ovaries were obtained from a local abattoir. Granulosa cells (Gc) and theca interna cells (Tc) obtained from the same follicles were subsequently prepared according to the technique described by Stoklosowa et al. [20, 21]. After isolation, Gc and Tc were collected and suspended in M199 medium supplemented with 10% fetal bovine serum. The viability of the cells was determined before seeding by the trypan blue exclusion test; viability was 60–75% for Gc and 85–90% for Tc. Gc and Tc were inoculated at concentrations similar to those observed *in vivo* (Gc : Tc, 4 : 1). The cultures were maintained at 37°C in a humidified atmosphere containing 5% CO_2_ for 24 h to allow for attachment. Then the cells were cultured for an additional 1, 6, 24, or 48 h with Halowax 1051 doses of 1, 10, and 100 pg/mL and 1 or 10 ng/mL.

### 2.3. Experimental Procedure

To determine CYP1A1 activity, cells were seeded in 96-well tissue culture plates at concentrations of 1.5 × 10^5^ viable cells per well and exposed to the test compound for 1, 6, 24, or 48 h. At the end of the incubation, media were removed and the cells were washed with cold phosphate buffered saline (PBS) and stored at −70°C.

To determine SULT1A and COMT activity, cells were seeded in 48-well tissue culture plates at concentrations of 2.5 × 10^5^ viable cells per well and exposed to the test compound for 6, 24, or 48 h. At the end of the incubation, media were removed and the cells were washed with cold PBS and stored at −70°C.

To examine the dose- and time-dependent effect of Halowax 1051 on CYP1A1, SULT1A, and COMT protein expression, cells were seeded in 24-well tissue culture plates at concentrations of 5.0 × 10^5^ viable cells per well and exposed to Halowax 1051 for 1, 6, or 48 h. After incubation, media were removed and the cells were washed with cold PBS and then lysed using Laemmli lysis-buffer. Total cell lysates were stored at −70°C.

### 2.4. CYP1A1 Activity

Frozen cells were lysed when removed from the freezer and allowed to thaw for 10 min. The ethoxyresorufin-O-deethylase assay (EROD), a specific measure of CYP1A activity, was performed as described by Kennedy and Jones [22]. The fluorescence of resorufin generated by the conversion of ethoxyresorufin by CYP1A was measured at 15 min interval for up to 2 h with a fluorescence plate reader (FLx 800, Bio-Tek, USA) using a 530 nm excitation filter and a 590 nm emission filter. After 2 h, the protein concentration in each well was determined by the fluorescamine protein assay (Sigma Chemical Co. MO, USA). Results were calibrated against a resorufin standard curve (0–100 nM) and a BSA standard curve (0–1000 *μ*g). 

### 2.5. COMT Activity

 COMT activity was measured using a modified colorimetric assay described by Herblin [23] (which uses methylation of 4-nitrocatecholas as a marker of COMT activity) with the following modifications. Frozen cells were thawed for 10 min. Each reaction well received 200 *μ*L of 25 *μ*M 4-nitrocatechol, 0.01 M of MgCl_2_, and 1 mM of Tris-HCl buffer (pH 7.0). Plates were preincubated for 10 min at 37°C. Then 0.2 mM of S-adenosyl methionine was added and incubated for an additional 60 min. The reaction was terminated by the addition of 1 M NaOH, and absorbance was measured at a wavelength of 520 nm using a micro-ELISA plate reader (Bio-Tek Instruments).

### 2.6. SULT1A Activity

SULT1A activity was measured using a modified colorimetric assay developed by Frame et al. [24]. This method is based on the release of p-nitrophenol from a 3′-phosphoadenosine-5′-phosphosulfate (PAPS) regenerating system. Frozen cells were thawed for 10 min. Each reaction well contained 50 mM of potassium phosphate buffer, 5 mM of MgCl_2_, 20 *μ*M PAPS, 5 mM p-nitrophenyl sulfate, and 0.1 mM 2-naphthol in a total volume of 250 *μ*L. As a negative control, cells were incubated for 48 h with the selective inhibitor of SULT1A, 2,6-dichloro-4-nitrophenol, at a dose of 0.5 *μ*M. The reactions were incubated at 37°C for 60 min and then terminated by the addition of 0.25 M of Tris-HCl buffer (pH 8.7). Absorbance was measured at a wavelength of 405 nm using a micro-ELISA plate reader (Bio-Tek Instruments).

### 2.7. Western Blot Analysis

Equal sample volumes were separated by SDS-PAGE and electrophoresed onto PVDF membranes using a Bio-Rad Mini-Protean 3 apparatus (Bio-Rad Laboratories, Inc., USA) according to the manufacturer instructions. Blots were incubated overnight with 1 : 200 dilutions of antibodies specific to CYP1A1 (sc-9828), SULT1 A (sc-27980), and COMT (sc-25844) (all from Santa Cruz Biotechnology Inc., CA, USA) and with 1 : 2000 dilution of antibodies specific to *β*-actin (A5316) (Sigma Chemical Co., MO, USA). An anti-*β*-actin antibody was used as a loading control. Primary antibodies were detected by a horseradish peroxidase-conjugated secondary antibody: P0447 (DakoCytomation, Denmark) for *β*-actin diluted 1 : 5000; sc-2020 (Santa Cruz Biotechnology Inc., CA, USA) for CYP1A1 and SULT1 diluted 1 : 2000; and sc-2004 for COMT diluted 1 : 2000, essentially according to the manufacturer's guidelines. Signals were detected by enhanced chemiluminescence using the Western Blotting Luminol Reagent (sc-2048, Santa Cruz Biotechnology) and were visualized using the ChemidocTM XRS+ System (Bio-Rad Laboratories). Data visualized by chemiluminescence were quantified by using Image LabTM 2.0 Software (Bio-Rad Laboratories).

### 2.8. Statistical Analysis

Each treatment was repeated three times (*n* = 3) in quadruplicate. Statistical analysis was performed using GraphPad Prism 5. Statistically significant differences between groups are indicated with different letters; the same letters indicating no significant differences, with a < b < c < d < e < f; statistically significant differences between control and treated groups were marked with *P < 0.05. All data (*n* = 12) are expressed as the mean ± the standard error of the mean.

## 3. Results and Discussion 

### 3.1. Phase I Metabolism

**Figure 1:**
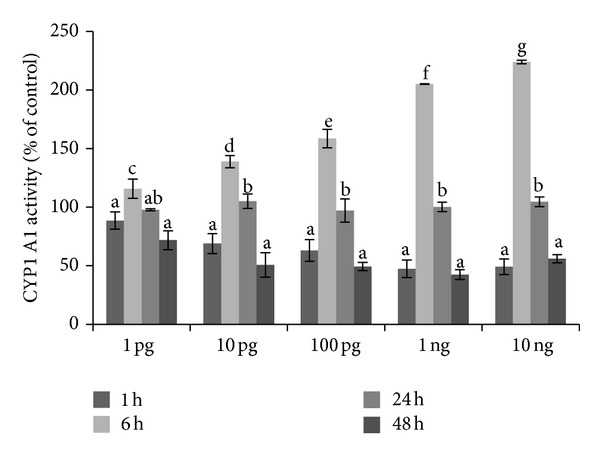
Time- and dose-dependent action of Halowax 1051 on CYP1 A1 activity showed as a percentage of control. Cocultures of granulose and theca cells were exposed to 1, 10, and 100 pg/mL and 1 or 10 ng/mL of Halowax 1051. Each treatment was repeated three times (*n* = 3). Statistically significant differences between points in graph are indicated with different letters; the same letters indicating no significant differences, with a < b < c < d < e < f < g.

**Figure 2:**
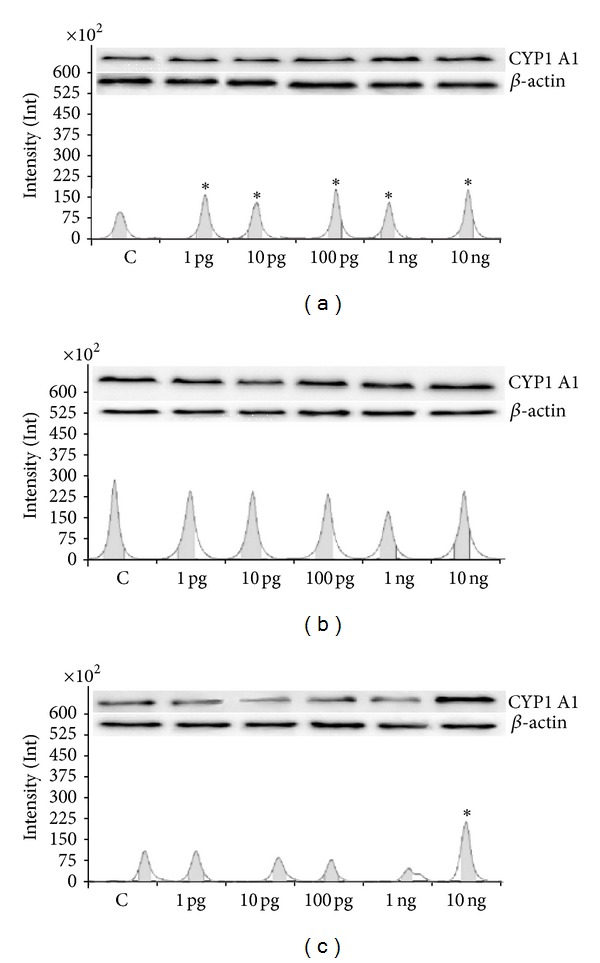
(a) Protein expression (immunoblot) of CYP1 A1 after 1 (a), 6 (b), and 48 (c) hours of incubation with Halowax 1051. The amount of protein in each sample was checked using an anti-*β*-actin antibody. All means marked with (*P < 0.05) are significantly different from the control.

CYP1A1 enzyme activity was assayed using the ethoxyresorufin-O-deethylase assay. Basal CYP1A1 activity was the highest in 6 h of incubation (with values: 26.27 ± 1.05; 101.08 ± 2.13; 16.36 ± 0.58; and 31.86 ± 0.93 pmol per 100 *μ*g protein min^−1^ after 1, 6, 24, and 48 h of culture, resp.). This is in accordance with our last published data [17] showing also the high basal CYP1A1 activity in ovarian follicles in 6 h of culture. A stimulatory effect on CYP1A1 activity was observed after 6 h of exposure to all doses of Halowax 1051 used (Figure 1). Basal CYP1A1 protein expression increased from 1 h to 24 h of culture and then decreased at the 48 h time point. Halowax 1051 after 1 h of exposure to all doses had a stimulatory effect on CYP1A1 protein expression. In addition, only for dose of 10 ng/mL activation of the CYP1A1 protein expression maintained for 48 h (Figure 2). Our previous studies have shown compound-dependent differences in the time of CYP1A1 activation: faster induction of the CYP1A1 protein by PCB3 (from 1 to 48 h) than by 17-*β* estradiol (from 6 to 48 h) [25].

The observed rapid activation of CYP1A1 under the influence of Halowax 1051 is probably associated with dioxin-like properties of PCN while longer-lasting activation under the influence of PCB-3 or 17-*β* estradiol with nondioxin-like properties of tested compounds. This suggests that the activation time depends not only on doses but also on type of used reagent.

Similarly, increased EROD activity in the livers of juvenile Baltic salmon, *Salmo salar*, exposed to a mixture of Halowax 1001, 1014, and 1051 [11] and in liver of rat exposed to PCNs [26] was demonstrated. Previously, Villeneuve et al. [27] estimated that relative potencies of individual PCNs in relation to a 2,3,7,8-tetrachlorodibenzodioxin standard generally increased with increasing chlorine substitution and were the highest for the most chlorinated compounds. Highly chlorinated PCNs are present in Halowax 1051. To our knowledge, this report is the first to show the impact of Halowax 1051 on microsomal enzymes in the ovary, suggesting that metabolites of PCNs are formed locally in this organ.

### 3.2. Phase II Metabolism

The second, and probably most important, finding was the inhibitory effect of Halowax 1051 on the activities and expression of COMT and SULT1A. The second phase of detoxification metabolism is particularly important because it leads to the formation of compounds that are removed via the urinary system. In the existing literature, no data show the detoxification of any PCNs by phase II enzymes in the ovaries. A very early study (by Cornish and Block) [28] showed that metabolites of hepta- and octachloronaphthalenes (main compounds of Halowax 1051) were absent from the urine of rabbits after the oral administration of PCNs, suggesting that the metabolism of these compounds proceeded only partially. 

**Figure 3:**
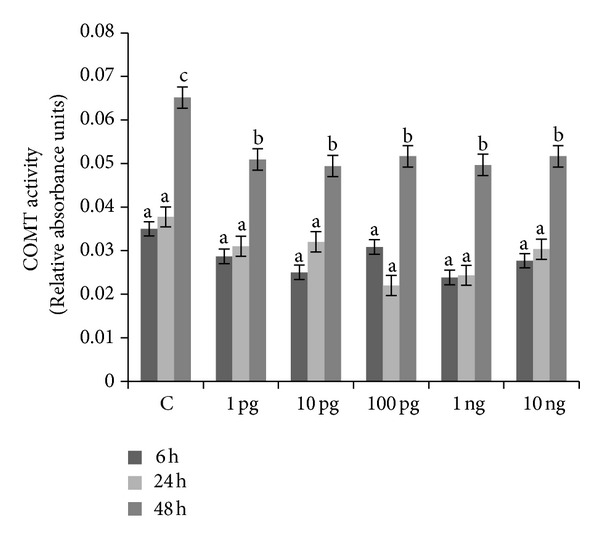
Time- and dose-dependent action of Halowax 1051 on COMT activity. Cocultures of granulose and theca cells were exposed to 1, 10, and 100 pg/mL and 1 or 10 ng/mL of Halowax 1051. Statistically significant differences between points in graph are indicated with different letters; the same letters indicating no significant differences, with a < b.

**Figure 4:**
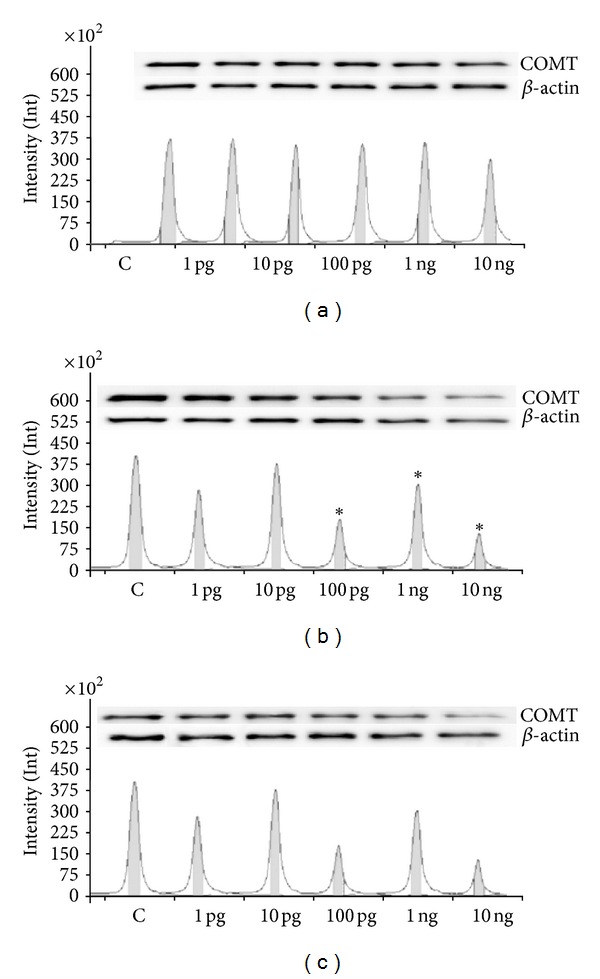
Protein expression (immunoblot) of COMT after 1 (a), 6 (b), and 48 (c) hours of incubation with Halowax 1051. The amount of protein in each sample was checked using an anti-*β*-actin antibody. All means marked with (*P < 0.05) are significantly different from the control.

 Basal COMT activities were 0.035 ± 0.003, 0.038 ± 0.004, and 0.065 ± 0.004 relative absorbance units after 6, 24, and 48 h, respectively. The inhibitory action of each dose of Halowax 1051 on COMT activity was noted after 48 h of exposure (Figure 3). A high level of COMT protein expression was observed in the control after 6 h of culture. COMT protein expression was decreased relative to the control after 6 h of exposure to 100 pg/mL, 1 ng/mL and 10 ng/mL, and after 48 h of exposure to 10 ng/mL of Halowax 1051 (Figure 4). 

 COMT is widely distributed throughout the animal kingdom and is primarily associated with the cytosolic fraction of many tissues including porcine granulosa cells [17, 29]. Substrates of COMT include xenobiotic catechols, catecholamines, and catechol estrogens. It has been shown that exposure to 2,2′,4,4′-tetrabromodiphenyl ether (BDE-47) increases COMT activity after 24 and 48 h with no effect on protein expression, as measured by immunoblot and ELISA analyses. This suggests that hydrophilic methoxylated polybrominated diphenyl ethers may be formed locally in the ovary. There are no previous data on the effects of Halowax 1051 on COMT activity in the ovary; however, Hernandez et al. [30] showed that COMT inhibition in pregnant rats produces arterial hypertension and endothelial dysfunction due to reduced nitric oxide bioavailability. Further, la Merril et al. [31] showed that exposure to 2,3,7,8-tetrachlorodibenzodioxin lowers COMT expression in mouse mammary glands, which are also hormone-dependent tissues. Therefore, the results in this paper are consistent with those of previous reports and indicate that hydrophilic methoxylated PCNs are not formed in the ovary.

**Figure 5:**
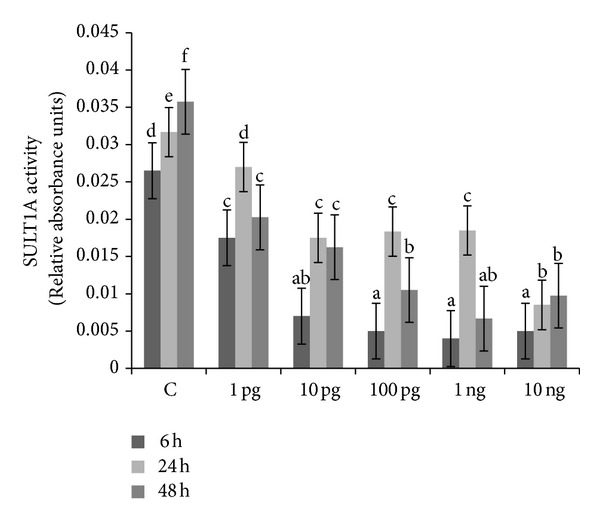
Time- and dose-dependent action of Halowax 1051 on SULT1A activity. Cocultures of granulose and theca cells were exposed to 1, 10, and 100 pg/mL and 1 or 10 ng/mL of Halowax 1051. Statistically significant differences between points in graph are indicated with different letters; the same letters indicating no significant differences, with a < b < c < d < e < f.

**Figure 6:**
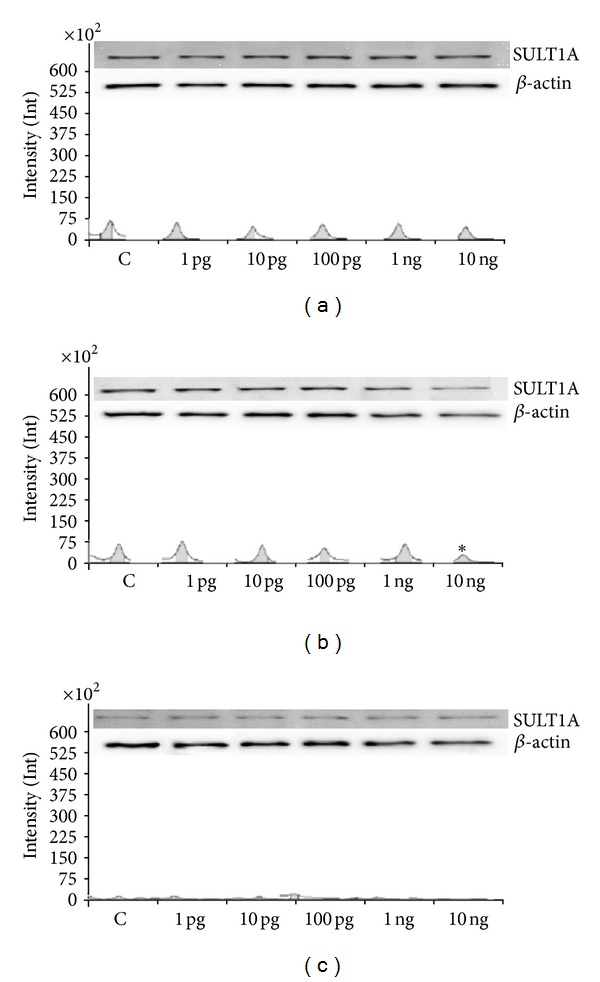
Protein expression (immunoblot) of SULT1A after 1 (a), 6 (b), and 48 (b) hours of incubation with Halowax 1051. The amount of protein in each sample was checked using an anti-*β*-actin antibody. All means marked with (*P < 0.05) are significantly different from the control.

 Basal SULT1A activities were 0.026 ± 0.007, 0.031 ± 0.006, and 0.035 ± 0.008 relative absorbance units after 6, 24, and 48 h, respectively. The inhibitory action of Halowax 1051 on SULT1A activity was noted at each dose and time point. The strongest effect of the mixture was observed after 6 h of exposure to doses ranging from 10 pg/mL to 10 ng/mL (Figure 5). Basal SULT1A protein expression was indistinguishable between 1 and 6 h of culture but decreased after 48 h of culture. SULT1A protein expression decreased after only 6 h of incubation with 10 ng/mL Halowax 1051 (Figure 6).

 SULT1A is the most abundant sulfotransferase; it has a broad substrate specificity and a wide tissue distribution [32]. Several SULT1A subfamily members contribute to sulfate conjugation of endogenous substrates, such as the thyroid hormone 17*β*-estradiol, as well as exogenous compounds including xenobiotics [33, 34]. Sulfate conjugation generally results in a decrease of biological activity and an increase in the hydrophilicity of compounds, which facilitates their excretion. As mentioned earlier, a previous study showed that rabbits could excrete 70 to 90% of ingested monochloro- or dichloronaphthalenes. Unfortunately, there have been no subsequent studies concerning PCN activation of enzymes involved in the second phase of detoxification metabolism. However, some studies have shown that other xenobiotics such as polychlorinated biphenyls may inhibit SULT1A activity [35]. As in the case of COMT activity, there have been no previous reports of the effects of Halowax 1051 on SULT1A activity in the ovary. Considering that sulfate conjugation of xenobiotics usually decreases their toxicity, we suggest that the inhibition of this pathway may lead to prolonged compound exposure in the ovary and subsequent disruption of ovarian function. 

## 4. Conclusion

The activation of phase I enzymes (CYP1A1) and inhibition of phase II enzymes (SULT1A and COMT) confirm the dioxin-like properties of PCNs. Fast activation of enzymes involved in phase I and concurrent inhibition of enzymes involved in phase II metabolism indicate that the observed effects of Halowax 1051 may partially result from the action of metabolites formed locally in ovarian follicles.
